# Future Basal Stem Rot, Oil Palm Mortality and Climate Scenarios for Oil Palm Compared to Climate Scenarios for Alternative Crops

**DOI:** 10.3390/microorganisms14030670

**Published:** 2026-03-16

**Authors:** Robert Russell Monteith Paterson

**Affiliations:** Centre of Biological Engineering, Gualtar Campus, University of Minho, 4710-057 Braga, Portugal; russell.paterson@deb.uminho.pt

**Keywords:** *Elaeis guineensis*, basal stem rot, *Ganoderma boninense*, soybean, maize, common bean, climate change, CLIMEX

## Abstract

Modifying food systems is required when they are threatened by a changing climate. Oil palm (OP) is a very important crop and climate change (CC) may decrease the areas in which OP can grow, as indicated by CLIMEX modelling. OP is affected by basal stem rot (BSR) and increasing incidences are indicated. Palm oil is used in many foods and biodiesel; Indonesia and Malaysia produce the largest volumes of the commodity. CLIMEX modelling of future suitable climates have also been applied to soybean, maize and the common bean (CB). The data for these crops were compared to those for OP in Indonesia, Malaysia and Thailand in the current paper to determine if growing the crops in the same regions in which OP is grown is possible in the future. Soybean had higher areas of suitable climate compared to OP. BSR and OP mortality further disadvantaged OP. The suitable climate for OP decreased significantly in Thailand by 2050 and in areas of Indonesia and Malaysia by 2070; the equivalent areas for soybean remained at high suitability. OP climate suitability further declined by 2100 in these and some other regions. Soybean could usefully be grown to diversify from the OP monoculture in many cases. Maize could be a possible alternative infrequently and the CB does not appear to be a viable alternative. These comparisons are unique and the methods could be employed in other systems.

## 1. Introduction

Maintaining the sustainability of the global food system is of fundamental importance. Disease, changing climate, weather extremes, population growth, environmental degradation and enduring poverty are all relevant to this issue [1]. Climate change (CC) is indisputable and related directly to greenhouse gas evolution largely from the burning of fossil fuels [2]. Furthermore, agricultural ecosystems in the tropics are threatened by climate variability, demographic pressures and resource-related issues [3] and are often less studied than those in temperate regions [4,5]. Ecosystems can be reshaped by climate thereby affecting the ability to grow crops. Diseases in crops affect crop sustainability and are also affected by CC.

Oil palm (*Elaeis guineensis*) (OP) is a monocot plant and a very important commodity system. Palm oil is added to numerous foods, drinks, cosmetics and biodiesel and is used for domestic cooking. However, the oil may not be included in supermarket foods as frequently as often stated and others may be added more, such as soy and maize [6]. Indonesia and Malaysia produce the most palm oil; Thailand, Papua New Guinea (PNG), Colombia and Nigeria are significant, but much lower, manufacturers [7]. The creation of OP plantations has involved deforestation and clearing of peat land which are detrimental to the environment and increase CC [8,9,10]. The OP system contributes to and is affected by CC [11,12,13].

CC contributes to (a) increased incidences of OP diseases and (b) reduced yields of palm oil and less OP growth [14] which may make OP unviable in some regions [11,12,13]. OP is affected by the major disease basal stem rot (BSR) caused by the fungus *Ganoderma boninense*, which is the correct species name despite the fungus being given numerous names previously [15]. The distribution of *G. boninense* is confined to Southeast Asia, Oceania, China and Japan and is (a) detected exclusively on OP and (b) projected to become more severe in the future under CC. The increase in incidence will be from decreased resistance of OP to CC and from the likelihood that a fungus, such as *G. boninense*, will adapt to novel conditions quicker than OP [11,12,13].

Palm oil has advantages over other vegetable oils. OP requires less land to provide equivalent amounts of oil and causes less environmental damage in that respect [16]. OP trees are very large and have the potential to sequestrate more damaging CO_2_ from the atmosphere which may assist in ameliorating CC [17]. The palm has a capacity for increased sustainable production without expansion into sensitive natural habitats. These characteristics do not consider sufficiently that the climate may not remain suitable for growing OP in the future. CLIMEX modelling of future suitable climates for growing OP [18,19] led to the novel method of granular inspection of the climate maps produced by such models, resulting in a series of papers about the effect of future climates on mortality and BSR in OP [11,12,13]. Murphy [16] suggested that OP may be more resistant to CC than other crops. On the other hand, Meijaard et al. [20] stated that OP plantations may face the greatest climate impact amongst oil crops, although the climate simulations available for major oil crops vary in scope and consistency, making comparisons difficult. Climate data need comparisons in a standardised manner for different crops to resolve this issue.

Procedures are required to ameliorate the effects of CC on OP [11,12,13] to assist the sustainability of the OP industry. Growing modified crops in regions with changed climates represents a method for adaptation. For example, modern agricultural systems rely heavily on cereal crops in India which are susceptible to climate stress, whereas millet is resilient to stress and offers a sustainable alternative [21]. *Glycine soja* is the wild relative of the cultivated soybean, *G. max* which is susceptible to various biotic and abiotic stresses. *G. soja* is climate resilient and could conceivably be employed to replace *G. max* [22]. Perennial crops such as OP have much greater potential for future food security and environmental sustainability than annual crops (e.g., soybean), although perennials are less frequently cultivated [23]. Cox et al. [23] propose that perennial varieties of annual crops should be developed. However, the effect of the future climate on these two types of crops has not been considered. An alternative may be to grow other crops which are already available. The climate for growing OP may deteriorate [11,12,13] and it would be useful to determine accurately the equivalent scenarios for other crops in the OP growing regions [24].

A negative impact on canola in Canada was determined by a model [25] and a similar scenario was posited for Europe [26]. The CC effects on sunflower cultivation determined that they may be favourable in northern Europe but there may be large declines in southern and eastern Europe [27]. The 2021 Global Gridded Crop Model Intercomparison [28] indicated that crop yield projections would involve (a) losses for maize, soybean and rice and (b) gains for wheat. Finally, large areas were expected to become unsuitable for soybean under CC scenarios, including the tropics [29].

CLIMEX modelling has been used extensively to provide scenarios for the effect of CC on various species [18] and generate climate maps. CLIMEX modelling of soybean [24] indicated that the climate would remain suitable in northern latitudes with losses in tropical areas, although southeast Asian regions appeared to retain high suitability. The tropics were projected to have decreased in suitable climates for maize, with an increase in poleward directions [30]. The climate for the common bean (*Phaseolus vulgaris*) (CB) was projected to decrease in Latin America and Africa and increase in Canada, the Nordic countries and Russia [31]. These are the only crops for which CLIMEX modelling was undertaken that used the same parameters as those for OP. However, the studies on soybean, maize and the CB did not focus specifically on areas where OP is grown commercially.

In the present report, CLIMEX modelling of the future climate for OP and BSR incidences in Indonesia, Malaysia and Thailand are determined. These are compared in a standardised manner with future climate scenarios for soybean, maize and the CB. Soybean is well known as a major competitor to OP [16] and maize oil is also included in many foods [6]. The CB would not generally be considered as an oil crop, although attempts have been made to produce oil from the commodity [32]. Only the data for CSIRO-Mk3.0 global climate model running SRES A2 are compared [18,19,24,30,31]. The information is used to determine if the crops could grow well in regions where OP is already grown. This represents the first time that such direct comparisons have been made in palm oil producing countries.

## 2. Materials and Methods

The climate maps in (a) Paterson et al. [18] for 2015, 2030, 2050, 2070 and 2100 and (b) Paterson et al. [19] for 2015, 2050 and 2100 were used for OP in the present paper. An average of the data for 2015 and 2100 was calculated. Only the data for CSIRO-Mk3.0 global climate model running SRES A2 were used. The maps were magnified on a computer screen using the well-known standard magnification facility provided by computers. The percentages of suitable climates were assessed visually to provide percentage suitabilities for growing OP as determined by the colours of the maps.

The distribution model for OP was developed using CLIMEX for Windows, Version 347 (Hearne Scientific Software Pty Ltd., Melbourne, Australia, 2007) under different climate scenarios, and climate data and CC scenarios were assessed using CliMond 10’gridded climate data. The potential future climate was characterised using A2 SRES scenarios, which are available from the CliMond dataset. The Global Biodiversity Information Facility was used for the fitting of the CLIMEX Parameters and the information on the global distribution of OP was used in parameter fitting. Southeast Asian distribution data were reserved for validation of the model. The OP distribution was determined by the Global Biodiversity Information Facility (GBIF) (http://www.gbif.org/, accessed 9 November 2015) and additional literature on the species in CAB Direct (http://www.cabdirect.org/web/about.html, accessed 9 October 2015), and the basis was formed for the collection of data regarding the distribution of OP. A mechanistic niche model using CLIMEX software supported ecological research incorporating the modelling of the potential distribution of species under differing climate scenarios and assumed that climate was the paramount determining factor of plant and poikilothermal animal distributions. The output from CLIMEX was categorised into areas and these were also based on other studies conducted through CLIMEX.

The Ecoclimatic Index (EI), which is scaled from 0 to 100, was used to weight the degree of suitability in the maps. Establishment of OP is only possible if EI > 0; 1–10 indicates marginal (syn. borderline) habitats, 10–20 is supportive of substantial populations, whilst > 20 is highly favourable. Thirty-three percent of the area on a map was calculated as suitable if the given part had marginal suitability. If a part of the map was given a substantial populations assessment, then 66% of the area was considered suitable. Finally, 100% of the area of the map was considered suitable when the map had a highly favourable assessment. The combined average areas were designated as the weighted suitable climate (WSC).

BSR and OP mortality in the three countries for the present paper were determined by adjusting published data [11,12,13] by a factor determined from WSC as follows:

**A1. BSR in Indonesia**:

1. F = WSC in reference [11] × WSC current paper

            100

2. BSR current = F × BSR in reference [11]

**A2. OP mortality (OPM) in Indonesia**:

1. F = WSC in reference [11] × WSC current paper

           100

2. OPM current = F × OPM in reference [11]

**BSR and OPM in Malaysia:**

The WSC and OP mortality for Malaysia were determined by taking the respective averages of the WSCs for Peninsular Malaysia, Sarawak and Sabah in [12]. These are designated WSCav and OPMav. The BSR incidences for the current paper were determined by:

**B1. BSR in Malaysia:**

1. F = WSCav × WSC current

        100

2. BSR current = F × BSR in reference [12]

**B2. OPM in Malaysia:**

1. F = WSCav × WSC current

      100

2. OPM current = F × OPMav

**C1. BSR in Thailand:**

1. F = WSC in reference [13] × WSC current

            100

2. BSR current = F × BSR in reference [13]

**C2. OPM in Thailand:**

1. F = WSC in reference [13] × WSC current

            100

2. OPM current = F × OPM in reference [13]

N.B. F = Conversion factor.

The climate maps provided for OP [18,19] were compared with those for the other core papers used in the present study for maize [30], soybean [24] and the CB [31] using the same global climate model CSIRO-Mk3 and the A2 future climate scenarios as used for OP. The maps for the maize, soybean and the CB papers were treated in the same manner as the OP maps.

The percentages of (a) areas of suitable climate, (b) BSR incidences and (c) OP mortalities were plotted using Excel. The trend lines were drawn using the Excel facility to give an estimation of the rate of change in suitable climate. Occasionally values of the trend line were greater than 100% which was a result of the mathematical extrapolation and did not represent a suitable climate value *per* *se*.

## 3. Results

### 3.1. Indonesia

**Figure 1:**
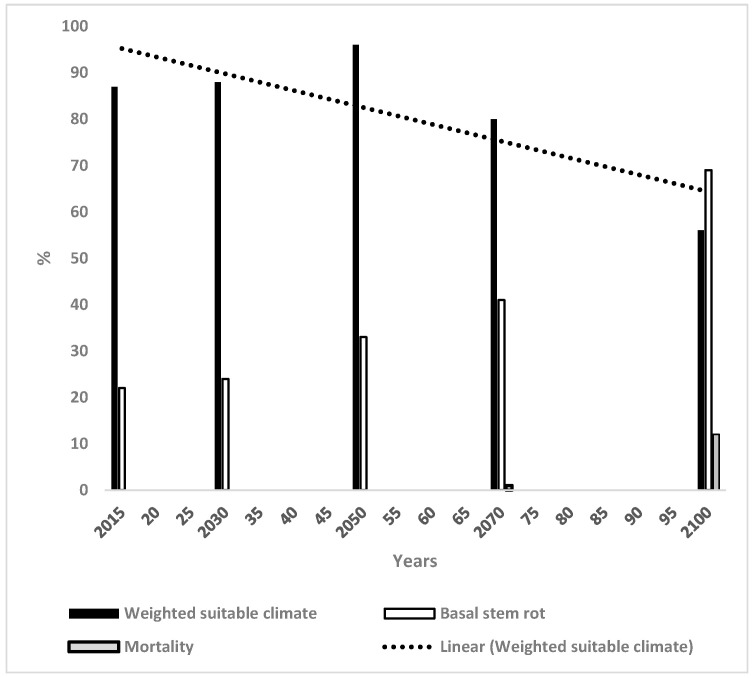
Change in weighted suitable climate (WSC), basal stem rot and oil palm mortality for Indonesia together with the trend line for WSC.

The WSC values for OP were 87% in 2015 which increased in a direct manner to 96% in 2050 and thereafter decreased to 80% and 56% in 2070 and 2100 respectively (Figure 1).

The trend line decreased from 96% in 2015 to 70% in 2100. The trend line from 2015 to 2070 decreased slightly from 90% to 83% from 2015 to 2100.

The incidence of BSR in OP in Indonesia (Figure 1) increased in an approximately direct manner from 21% to 72% from 2015 to 2100 respectively. The mortality of OP from climate effects was zero until 2070 at which time it was 1% and then increased to 10% by 2100.

**Figure 2:**
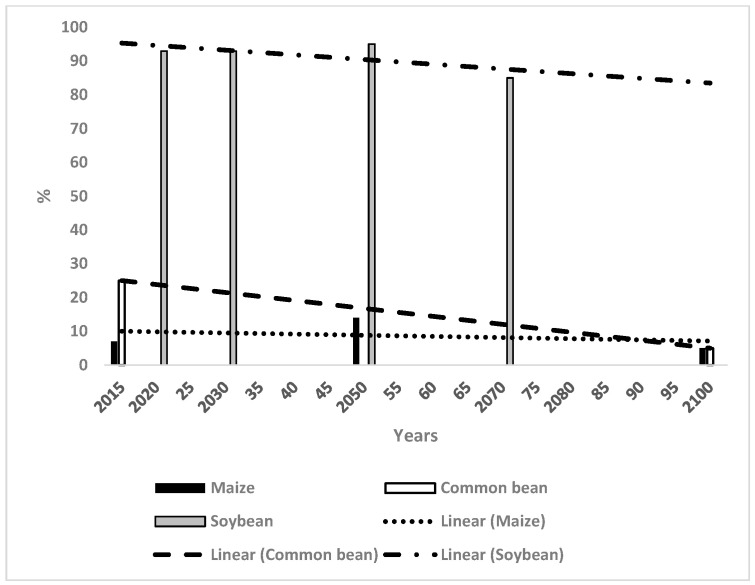
Change in weighted suitable climate in Indonesia for maize, the common bean and soybean and the relevant trend lines.

The WSC values for maize and the CB were much lower than those for OP and soybean (Figure 2), with 7% in 2015 for maize which increased to 14% in 2050 and then fell to 5% in 2100. The trend line was horizontal at 10% throughout. The CB’s values were at 25% in 2015, decreasing to 5% by 2100, which represented the same values as the trend line. The WSC figures for soybean were 93% in 2020 and 2030 which increased to 95% in 2050 and decreased to 85% in 2070 (Figure 2). The trend line was 96% in 2015, decreasing to 90% by 2100.

### 3.2. Malaysia

**Figure 3:**
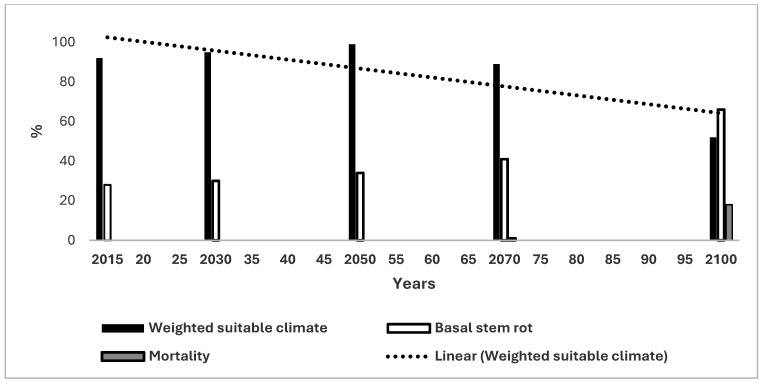
Change in weighted suitable climate (WSC), basal stem rot and oil palm mortality for Malaysia together with the trend line for WSC.

The change in WSC for OP in Malaysia is provided in Figure 3. WSC was 91% for OP in 2015 which increased in a direct manner to 99% in 2050 and then decreased to 89% and 52% in 2070 and 2100 respectively. There was a distinctly decreasing trend line from 103% in 2015 to 65% in 2100. Clearly, the 103% value did not represent climate suitability *per se* but was the consequence of the mathematical extrapolation used to give the slope to the trend line (see Methods). The trend line using the 2015 to 2070 data was horizontal at a value of 95%. There was an approximately direct increase in BSR in Malaysia from 25% to 70% from 2015 to 2100 respectively (Figure 3). The mortality of OP from an inclement climate was 0% until 2050, which increased to 1% and 10% in 2070 and 2100 respectively.

**Figure 4:**
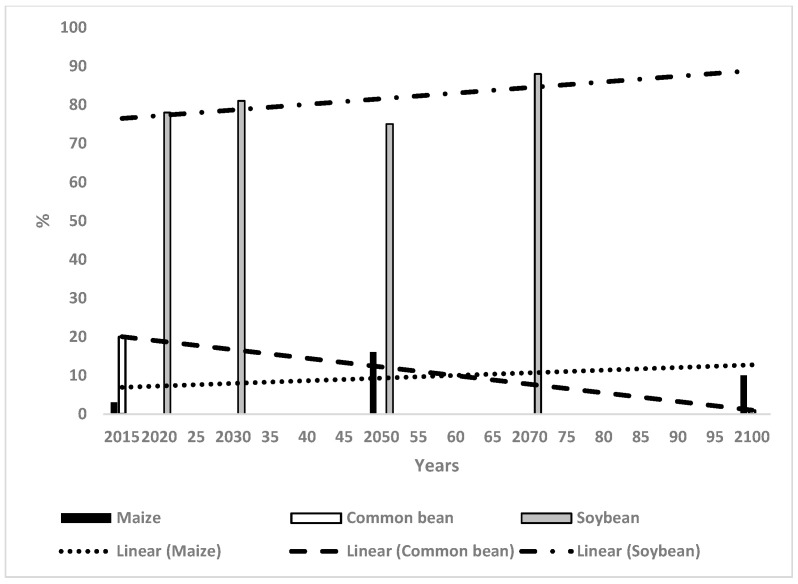
Change in weighted suitable climate in Malaysia for maize, the common bean and soybean and the relevant trend lines.

The WSC values for maize were 2%, 16% and 10% in 2015, 2050 and 2100 respectively, resulting in a slightly increasing trend line from 5% to 12% in 2015 and 2100 respectively (Figure 4). The values for the CB were 20% and 1% in 2015 and in 2100 respectively, resulting in a trend line with the same values (Figure 4). The change in WSC for soybean was 78% in 2020, increasing to 81% by 2030, which then decreased to 75% in 2050, followed by a significant increase to 88% by 2070 (Figure 4). This resulted in a distinctly increasing trend line from 78% in 2015 to 92% in 2100.

### 3.3. Thailand

**Figure 5:**
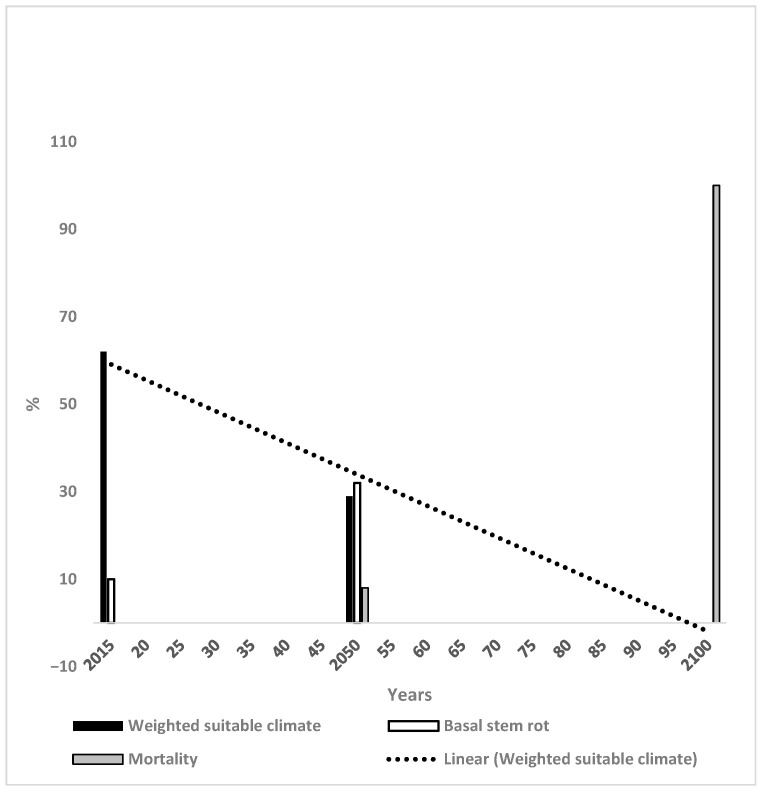
Change in weighted suitable climate (WSC), basal stem rot and oil palm mortality for Thailand together with the trend line for WSC.

**Figure 6:**
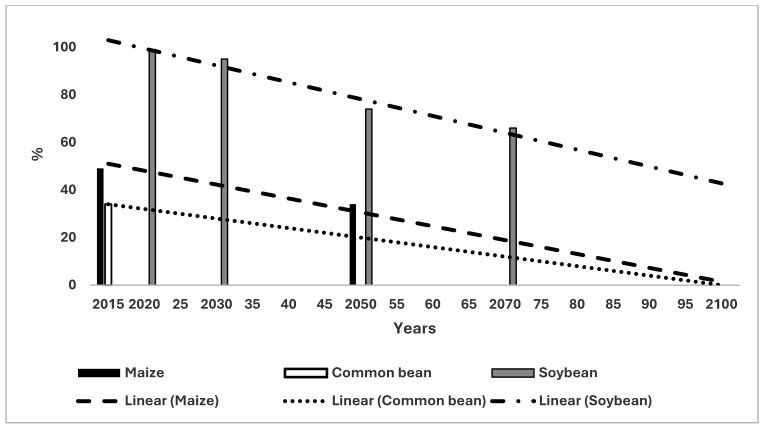
Change in weighted suitable climate in Thailand for maize, the common bean and soybean and the relevant trend lines.

The WSC values for OP in Thailand were 62%, 29% and 0% in 2015, 2050 and 2100 respectively (Figure 5). The trend line was 60% in 2015 and −2% by 2100. The incidence of BSR was 9% and 32% in 2015 and 2050 respectively in Thailand (Figure 5). In 2100, mortality was determined as 100% and there would be no OP because of the unsuitable climate. OP mortality was 6% in 2050. The WSC for soybean, maize and the CB in Thailand are demonstrated in Figure 6. The WSC changes for maize were 48% in 2015 decreasing to 40% and 0% in 2050 and 2100 respectively (Figure 6). The trend line was 52% in 2015, decreasing to 0% by 2100. For the CB, the value of 2015 was 34%, decreasing to 0% by 2100. The trend line had the same values. Soybean had values for WSC for 2020 and 2030 of 99% and 95% respectively, which decreased to 74% and 66% by 2050 and 2100 respectively. The trend line changed from 103% in 2015 to 68% by 2100. As mentioned, the 103% value did not represent climate suitability *per se* but was the consequence of the mathematical extrapolation to give the slope to the trend line (see Methods).

## 4. Discussion

The information on future suitable climates for growing OP is compared to equivalent data for three other crops using standardised methods in the current paper. The derived data on BSR and mortality of OP are also provided. The data on the mortality of OP are from the effect of an inclement climate but there would also be an estimated 50% mortality of OP from BSR infection [33], which is not included in the OP mortality determined herein.

Table 1 provides a qualitative summary of the initial climate suitability for the four crops in each country and the trends of WSC based on Figure 1, Figure 2, Figure 3, Figure 4, Figure 5 and Figure 6. The climate for soybean is more favourable than that for OP in general for the three countries. Maize and the CB do not appear capable of competing with OP as an alternative crop in most cases. The authors of reference [34] stated that the current trend of producing alternative vegetable oils to palm oil is partially a response to the perceived detrimental effects of OP on the environment. Replacing palm oil would have an insignificant effect on global GHG emissions whilst causing an increased risk of deforestation. The scenarios presented in the present paper indicate that soybean may have a more suitable climate for growth than OP in the future.

There is an already high incidence of BSR in OP in the three countries under consideration which will reduce yields and cause an estimated increased mortality of OP of 50% [33], in addition to the mortality and yield loss from the increasingly unfavourable climate. The incidence of BSR progressed to very high incidences by 2100 under the current schemes.

### 4.1. Soybean in Relation to Oil Palm

Soybean could replace OP due to the advantages that might accrue from diversification away from the OP monoculture. It is well known that crop monocultures are susceptible to disease or an inclement climate causing crop failure.

#### 4.1.1. Indonesia

There are high incidences of BSR in OP in Indonesia by 2070 under the current assessment. Most of Indonesia had similarly high suitable climates for soybean as those for OP until 2070 (Figure 1), the latest time for which there were data for both crops. The added stress of BSR makes soybean an even more attractive possible alternative to OP by 2070. Of course, soybean will also experience fungal and other diseases [35], although new plantings of soybean would have low disease incidence compared to the high BSR incidence of OP currently.

The WSC from 2050 to 2070 for OP decreased from 96% to 80%, i.e., 16%, compared to a decrease from 95% to 85% for soybean, i.e., 10%. Hence, the WSC for soybean decreases 6% less than that for OP during this period which slightly favours soybean. However, the incidence of BSR for OP was 41% in 2070 (Figure 1), making the overall situation much less favourable for OP than that for soybean.

The suitable climate for soybean was demonstrably higher than that for OP in Java and the Lesser Sunda islands by 2070 (Table 2) which represents the earliest year for this to occur in Indonesia. In addition, BSR in Java was at high incidence [11] which represents another negative influence on the sustainability of the OP industry. Soybean could be grown in these regions to complement the OP crop and to protect against failure of OP due to CC and BSR. These regions become much less suitable for OP by 2100 (Table 2; [18,19]) which supports the general premise. The reduced suitability of the climate for OP spread to Sumatra, Kalimantan and Papua by 2100 and an intrinsically unsuitable climate was determined for north Java, south Sumatra and west Kalimantan [18,19]. The BSR assessments for Kalimantan and Sumatra indicated a high incidence, whereas the disease was at a low incidence in Papua [11]. The sustainability of the OP industry in these regions by 2100 would be low, whereas the climate suitability for soybean would be high; soybean may be a suitable complementary crop to OP in large areas of Indonesia, if these scenarios are proven correct.

#### 4.1.2. Malaysia

BSR incidence of OP in Malaysia was 41% and OP mortality from an inclement climate was projected as 1% in 2070. BSR infection is considered to cause 50% OP mortality (see above); hence, the overall environment for OP would be highly disadvantageous by 2070. As mentioned, soybean would also be subjected to disease, but it would be at a low level, whereas BSR in OP is at a high incidence currently. In addition, there is the pressure of unsuitable climates for growth as discussed herein. Most of Malaysia had similarly high suitable climates to soybean and OP until 2070, the latest time for which there were data available for both crops. However, between 2050 and 2070 in Malaysia the suitability for OP decreased 10% from 99% to 89%. The equivalent figure for soybean was an increase of 13% from 75% to 88%. This represents a highly significant difference of 23% and indicates that the suitable climate for soybean in 2100 may decrease less than the 52% value determined for OP (Figure 3 and Figure 4). Soybean may be a useful replacement for OP in some cases on the basis.

The Malaysian OP industry originated in the west coast of peninsular Malaysia where many plantations are currently located [36]. The region was determined to have only a marginal climate for OP by 2070, a decrease from a highly suitable climate (Table 2). The west coast of peninsular Malaysia became unsuitable *per se* in 2100 for OP. Parts of Sarawak, Malaysia also have marginal climate suitability, in this case by 2100. The same areas were designated as having a highly suitable climate for soybean [24]. Hence, there is a convincing argument for diversification into soybean for these regions of the country, which is supported by BSR being projected as being at high incidence in the west coast of peninsular Malaysia and somewhat supported for Sarawak, although BSR had a low incidence in this case [12].

The only region of the three countries under consideration where soybean had a low level of suitable climate was the marginal designation of central Sarawak in Malaysia [24]. OP had high suitability in this region. Diversification into soybeans is clearly not indicated in this small area.

#### 4.1.3. Thailand

The results are obvious particularly for Thailand. BSR incidence is at a high level in Thailand currently (Figure 5) [13]. The climate for the country will be 100% unsuitable for OP by 2100 implying that it would be impossible to grow OP. The climate is much more suitable for soybean than OP in Thailand (Figure 5 and Figure 6), and soybean is without the problems of a particular disease compared to the situation for OP. Thailand had a significant loss of suitability for OP by 2050 due to marginal climate which represents the earliest such decline of the three countries considered. The high WSC for soybean covers most of Thailand from 2020 until 2070, with two pockets of marginal climates toward the northwest and southwest by 2070 [24]. Marginal climate for OP covers most of the country by 2050 with considerable amounts of unsuitable climates in the west of the country [19]. Even the small percentage of highly suitable climates for OP in the south of the country was replaced by unsuitable climates by 2100. Hence, soybean could be considered as a replacement for OP, if the future suitable climate and BSR incidence described in the present paper occur.

Cox et al. [23] mentioned that perennial crops have much greater potential for future food security and environmental sustainability than annual crops (e.g., soybean). However, the current scenarios indicate that the future suitable climate for growing soybean is much higher generally than that for OP indicating a reduced level of food security for OP. The environmental sustainability of soybean under these circumstances would require assessment.

##### Maize in Relation to Oil Palm

The initial climate suitability for maize was low in Malaysia and Indonesia and at a medium level in Thailand. Maize does not offer a particular advantage over OP in most regions, but it may be worth considering for diversification in a limited sense. There were moderately high levels of WSC for maize in Sulawesi, Indonesia until 2100 (Figure 2), although OP also had high suitability (Table 2). BSR in OP was at a low level in Sulawesi [11] indicating that the sustainability of OP could be maintained until 2100. Nevertheless, maize could perhaps be grown to complement OP. A significant region in north Sumatra had moderately high WSC for maize in 2050 and 2100 [30]. This area could perhaps be considered for more maize production as the region also had a low suitable climate for OP in 2100 (Table 2, [37]). The two regions mentioned above also have high WSC for soybean which could also be a crop used for diversification [24]. There was marginal WSC for maize in the north of peninsular Malaysia from 2015 until 2100 with no particular benefit over OP. The suitable climate for maize had moderate values in Thailand and could be considered as an alternative to OP in a limited sense after 2050. In all these cases, the threat from BSR requires consideration as it makes OP more vulnerable to future climates and provides a reason for diversification into other crops. Maize has associated diseases, which is well known, but newly planted maize may have fewer diseases than OP whereas BSR would already be at a high level.

##### The Common Bean in Relation to Oil Palm

The WSC for the CB decreased with time and seldom represented an obvious replacement for OP. The WSC for the CB was quite high in Sulawesi [31] and it may represent a diversification possibility from OP. Also, the WSC for the CB had moderately high values in Thailand after 2050, where it could conceivably provide an alternative to OP together with maize and soybean. The threat from BSR to OP also requires consideration, as described above.

There are increasing reports of *G. boninense* having the potential to produce mycotoxins from metabolomic [38] and genetic analyses [39,40]. Mycotoxins are secondary metabolites produced by a limited number of fungi that contaminate food and cause severe illness in humans and occasionally death [41]. The methods employed in [38,39,40] would not detect the compounds if they were produced by the fungus. It is unfortunate that the authors did not analyse the strains of mycotoxins because production appears crucial to their general hypotheses. The metabolites could be translocated into palm oil when the fungus infects OP and health problems in humans could arise when the commodity is consumed. Indeed, mycotoxins have been detected in palm oil and are of concern [42]. There is an urgent requirement for a systematic programme of research involving the detection of mycotoxins from *G. boninense* to resolve this issue. Scientific procedures and criteria for testing mycotoxin production from fungi have been published and could be adapted for *G. boninense* [43].

Furthermore, a recent paper claimed that *G. ellipsoideum* causes BSR in OP [44]; although, the details of the plant from which the fungus was isolated were ambiguous and evidence of pathogenicity was not provided. The fungus was considered closely related to *G. boninense* despite the two species being different [15]. Lakshmi et al. [45] provided details of *G. ellipsoideum* being isolated from a diseased OP which infected the palm in vitro. It is remarkable that two species can cause the same disease, and further investigations are required to determine if *G. ellipsoideum* does in fact cause the disease in India and elsewhere.

The suitable climate for growing OP was projected to increase dramatically from 2015 to 2100 in certain elevated regions of Indonesia and Malaysia [46]. The threat of BSR would be considered low if OP was planted in many of these regions because the plantings would be new and without a history of BSR. The suitability of the climate for growing soybean was also at high level in these regions, except for a small zone in central Sarawak [24], as mentioned above. Soybean could also be grown in addition to OP to gain the advantages of diversification.

The major conclusion of this current paper is that the future suitable climate of soybean is more favourable than that of OP in OP growing regions. The conditions for maize and the CB are much less favourable. Murphy [16] and Meijaard et al. [20] had differing opinions to each other on the susceptibility of OP to CC from scenarios (see Section 1), but here the situation appears to be that soybean is more resistant. The large increases in the incidence of BSR on OP add additional stress to the OP food system. The current results indicate that OP could conceivably be replaced by soybean, especially where the climate becomes unsuitable or marginal for OP. Soybean could be grown adjacent to, or instead of, OP as a form of diversification and would become productive rapidly compared to the long time periods involved in the maturation of OP. Diversification of crops is well known to provide advantages over monocultures [47] especially with respect to failure of monoculture crops from, for example, disease or severe climate.

Finally, the three countries discussed herein have already introduced soybean, maize and the CB (Table 3) and the knowledge exists to grow these crops.

## 5. Conclusions

The major conclusion of this paper is that the future climate of soybean is more suitable than that for OP in three major OP growing countries. BSR in OP was at a high incidence initially and increased rapidly in the three countries as the suitable climate for OP decreased until 2100. The industry will experience severe problems of sustainability in the future if these scenarios prove accurate in reality. The trends for soybean were more complex with an increasing trend for Malaysia, a slightly decreasing trend for Indonesia and a large decreasing trend for Thailand (Figure 1, Figure 3 and Figure 5). Soybean had a highly suitable climate until 2070 and could be a viable replacement for, or complement to, OP. Finally, it would be necessary to determine the socio-economic feasibility, land-use implications and deforestation risks of planting soybean to compensate for losses of OP. The trends for the other crops were less advantageous when acting as replacement crops for OP.

Modelling does not provide accurate predictions for future climates. The results of modelling provide scenarios from which it is possible to make plans to mitigate the negative effects of CC which can be modified as real time data emerges [49,50]. The procedures used in the present paper allow comparisons of climate maps for different crops which would be difficult to undertake by visual inspections of the maps alone. More modelling studies for OP are required. Finally, the methods presented may be useful for climate maps in general and can be obtained by other modelling techniques.

## Figures and Tables

**Table 1:** Initial degree of suitability of climate for the four crops and the trend in suitability until (a) 2100 for oil palm, maize and the common bean and (b) 2070 for soybean.

Country		Oil Palm	Soybean	Maize	Common Bean
**Indonesia**	*Initial suitability*	High	High	Low	Medium
	*Trend to 2100*	Large decrease	Slight decrease	No change	Large decrease
**Malaysia**	*Initial suitability*	High	High	Low	Medium
	*Trend to 2100*	Large decrease	Increase	Slight increase	Large decrease
**Thailand**	*Initial suitability*	Medium	High	Medium	Medium
	*Trend to 2100*	Large decrease	Large decrease	Large decrease	Large decrease

**Table 2:** Areas with marginal and unsuitable climate for growing oil palm by 2050, 2070 and 2100 [18,19] and where suitability is high for soybean. N.B. there are no soybean data for 2100.

Country	2050 Marginal	2050 Unsuitable	2070 Marginal	2100 Marginal	2100 Unsuitable
*Indonesia*			1. North Java. 2. Lesser Sunda Islands.	1. Most of Java. 2. Lesser Sunda Islands. 3. Most of Sumatra. 4. Large area of south Kalimantan. 5. Large area of south Papua. 6. Small area of south Sulawesi.	1. North Java. 2. Substantial areas of south Sumatra. 3. Substantial areas of west Kalimantan.
*Malaysia*			West coast Peninsular Malaysia	1. Most of Peninsular Malaysia. 2. South Sarawak.	West coast Peninsular Malaysia
*Thailand*	Very large proportion.	Substantial part of west			Almost all

**Table 3:** Regions where soybean, maize and the common bean have been introduced in the three countries [48].

		CROP	
Country	Soybean	Maize	Common Bean
**Indonesia**	Java, Papua	Kalimantan, Papua, Java	Java, Papua
**Malaysia**	North peninsula Malaysia	Almost all in parts	Most of peninsular Malaysia
**Thailand**	Widely	Almost all in parts	Most in parts

## Data Availability

The original contributions presented in this study are included in the article. Further inquiries can be directed to the corresponding author.

## Abbreviations

The following abbreviations are used in this manuscript:

CC	Climate change
OP	Oil palm
OPM	Oil palm mortality
